# Theoretical and Experimental Investigation of the Antioxidation Mechanism of Loureirin C by Radical Scavenging for Treatment of Stroke

**DOI:** 10.3390/molecules28010380

**Published:** 2023-01-02

**Authors:** Ye-Shu Liu, Guo-Ying Zhang, Yue Hou

**Affiliations:** 1 College of Life and Health Sciences, National Frontiers Science Center for Industrial Intelligence and Systems Optimization, Northeastern University, Shenyang 110819, China; 2 Key Laboratory of Data Analytics and Optimization for Smart Industry, Northeastern University, Ministry of Education, Shenyang 110819, China; 3 College of Physics Science and Technology, Shenyang Normal University, Shenyang 110034, China

**Keywords:** loureirin C, OH^•^ radical scavenging, antioxidation mechanism, DFT calculations, the most preferred hydrogen abstraction mechanism

## Abstract

Recent pharmacological studies have shown that dragon’s blood has an anti-cerebral ischemia effect. Loureirin C (LC), a kind of dihydrochalcone compound in dragon’s blood, is believed to be play an important role in the treatment of ischemia stroke, but fewer studies for LC have been done. In this paper, we report the first experimental and theoretical studies on the antioxidation mechanism of LC by radical scavenging. The experimental studies show that LC has almost no effect on cell viability under 15 μM for the SH-SY5Y cells without any treatments. For the SH-SY5Y cells with oxygen and glucose deprivation-reperfusion (OGD/R) treatment, LC increased the viability of SH-SY5Y cells. The results of 2′,7′-Dichlorodihydrofluorescein diacetate (DCFH-DA) and MitoSox Red experiments indicate that LC is very efficient in inhibiting the generation of the intracellular/mitochondrial reactive oxygen species (ROS) or removing these two kinds of generated ROS. The density functional theory (DFT) calculations allowed us to elucidate the antioxidation mechanisms of LC. Fukui function analysis reveals the radical scavenging of LC by hydrogen abstraction mechanism, the complex formation by e-transfer, and radical adduct formation (RAF) mechanism. Among the H-abstraction, the complex formation by e-transfer, and radical adduct formation (RAF) reactions on LC, the H-abstraction at O-H35 position by OH^•^ is favorable with the smallest energy difference between the product and two reactants of the attack of OH^•^ to LC of −0.0748 Ha. The bond dissociation enthalpies (BDE), proton affinities (PA), ionization potential (IP), proton dissociation enthalpy (PDE), and electron transfer enthalpy (ETE) were calculated to determine thermodynamically preferred reaction pathway for hydrogen abstraction mechanism. In water, IP and the lowest PDE value at O3-H35 position are lower than the lowest BDE value at O3-H35 position; 41.8986 and 34.221 kcal/mol, respectively, indicating that SEPT mechanism is a preferred one in water in comparison with the HAT mechanism. The PA value of O3-H35 of LC in water is −17.8594 kcal/mol, thus the first step of SPLET would occur spontaneously. The minimum value of ETE is higher than the minimum value of PDE at O3-H35 position and IP value, 14.7332 and 22.4108 kcal/mol, respectively, which suggests that the SEPT mechanism is a preferred one in water in comparison with the SPLET mechanism. Thus, we can draw a conclusion that the SEPT mechanism of is the most favorite hydrogen abstraction mechanism in water, and O-H35 hydroxyl group has the greatest ability to donate H-atoms.

## 1. Introduction

Stroke has received widespread attention because of its substantial morbidity and disability [1]. Stroke can be divided in two different categories: ischemic and hemorrhagic. Ischemic stroke is characterized by vascular occlusion that causes inadequate perfusion of oxygenated blood and depleted supply of nutrients to the brain. Even when blood flow is restored, secondary damage caused by reperfusion can be observed in cerebrovascular system and neural networks. The mechanism of the pathological process of ischemic stroke is very complicated and unclear. In recent years, studies have found that ferroptosis [2], oxidative stress, inflammation, and excitotoxicity are the underlying mechanisms of brain damage in ischemic stroke [3]. Ferroptosis is a novel form of programmed cell death in which the accumulation of intracellular iron promotes lipid peroxidation, leading to cell death [2,4]. Excess intracellular free Fe^2+^ deposition will initiate the Fenton reaction to generate ROS (Fe^2+^ + H_2_O_2_ + H^+^ → Fe^3+^ + OH^•^ + H_2_O) [5]. The chain-reaction process of lipid peroxidation, which can be induced by a free-radical source, continually produces lipid peroxide radicals. On the one hand, the excessive ROS can stimulate the expression of cytokines and adhesion molecules, resulting in inflammation and immune response [6,7]; on the other hand, excessive ROS can react with proteins and nucleic acids, which causes cellular apoptosis and cell death in the brain [8]. Thus, after stroke, lipid peroxidation is considered to be one of the basic mechanisms involved in cell and tissue damage [9,10]. Thus, the neuroprotective therapy is a main strategy for the treatment of ischemic stroke by scavenging the free radicals and suppressing oxidative stress [11,12,13], and it is significant to find antioxidants for scavenging the excess ROS and then repairing the oxidative damage in the brain caused by ischemic stroke.

It is well known that polyphenolic flavonoids found in many foods and medicinal plants have beneficial effects on the human health, which attributes to their antioxidant and radical scavenging properties [14,15]. For example, *trans*-resveratrol can break the chain-reaction process of lipid peroxidation by scavenging free radicals, which results in the suppression of harmful self-propagating reactions. Dragon’s blood, with Chinese name “Longxuejie”, is a Chinese herbal medicine [16]. Studies have shown that dragon’s blood contains phenols, terpenoids, steroids, steroid saponins, and other components. The main chemical components of dragon’s blood are phenolic compounds which are the main physiological active components [17]. Phenolic compounds mainly contain flavonoids and stilbenoids. According to their structures, flavonoids can be divided into several classes, e.g., chalcone, dihydrochalcone, flavone, flavane, polyflavone, and the other phenolic compounds. Dihydrochalcone is a relatively rich component in dragon’s blood, it contains loureirin A, loureirin B, loureirin C, loureirin D, etc. Recent pharmacological studies have shown that dragon’s blood has antithrombotic [18], anti-cerebral ischemia [19], anti-inflammatory, anti-diabetic, analgesic, and radio-protective activities. In addition, dragon’s blood also has the effect of enhancing immune function, and it can promote blood circulation and stop bleeding [20]. Therefore, it is believed that some components of dragon’s blood can be used in the treatment of ischemic stroke. He et al. [21] demonstrated that resveratrol, a phenolic compound in dragon’s blood, protected against cerebral ischemia/reperfusion(I/R) injury in rats by inhibiting NLRP3 inflammasome activation through Sirt1-dependent autophagy activity. Pterostilbene, also a phenolic compound in dragon’s blood, showed a protective function on both neurons and cerebral tissues against ischemic stroke injuries by modulating micro-ribonucleic acid (miR)-21-5p/Programmed Cell Death Protein 4 (PDCD4) axis in vivo and in vitro [22]. While fewer studies of the dihydrochalcone in dragon’s blood have been done for the treatment on ischemic stroke, especially LC has not been addressed for this disease treatment. Therefore, the main goal of the present work is to perform a detailed study on the free radical scavenging properties of LC and to reveal the underlying mechanisms.

In the past few decades, density functional theory (DFT) has been widely applied for predicting the biological activity and revealing its mechanism of action of some drugs [23,24,25], which has become a valuable technique to determine and design and highly active drug molecules. The greenness of this theoretical technique lies in the fact that it provides the lots of useful information with its efficiency and reasonable accuracy, and without causing harm to the environment. Unambiguously, a combination of theoretical and experimental studies can lead to a better understanding of the mechanism of action of some drugs. 

Here, we reported the preparation method of LC. Furthermore, the cytotoxicity of LC against SH-SY5Y cell and SH-SY5Y cell treated with OGD 6 h followed by 22 h reperfusion was evaluated. Then, the effects of LC on the intracellular ROS and mitochondrial ROS were investigated by the DCFH-DA staining assay and MitoSox staining assay. For theoretical studies, we first determined the preferred conformation of LC by DFT theory calculations. Furthermore, we modeled LC reactions with OH^•^ radical in gas phase and water environments to predict the possible mechanisms for the radical scavenging. Three different hydrogen atom abstraction reaction mechanisms were considered to determine the most favorite mechanism in gas phase or in water.

## 2. Results and Discussion

### 2.1. The Experimental Studies of the Antioxidant Activities of LC 

#### 2.1.1. Evaluation of LC on Neuron Viability

**Figure 1:**
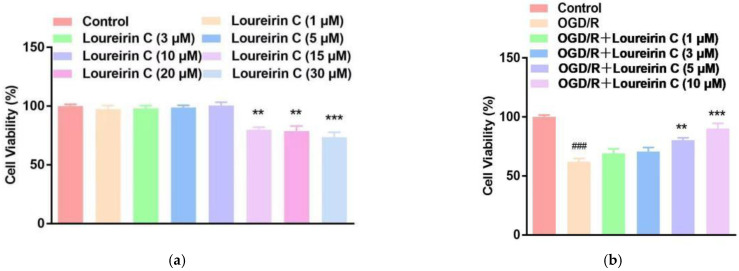
LC attenuated OGD/R-induced neuronal injury in vitro. (**a**) The viability of the SH-SY5Y measured by MTT. (**b**) The viability of the SH-SY5Y with OGD/R treatment measured by MTT. Data are expressed as mean ± SD (*n* = 3). *** * p* < 0.001, ** *p* < 0.01 vs. Control group in (**a**); *** *p* < 0.001, ** *p* < 0.01 vs. OGD/R group, and ### *p* < 0.001 vs. Control group in (**b**).

We initially evaluated the cytotoxicity of LC on the SH-SY5Y cells by 3-(4,5-dimethylthiazol-2-yl)-2,5-diphenyltetrazolium bromide (MTT) assay. The SH-SY5Y cells were exposed to LC at the concentrations of 1, 3, 5, 10, 15, 20, and 30 μM for 24 h. As shown in Figure 1a, LC had almost no effect on cell viability under 15 μM. Thus, 10 μM or less of LC was chosen in the following assays. Subsequently, we performed the OGD/R model in SH-SY5Y cells (OGD 6 h followed by 22 h reperfusion) to investigate the brain protective effect of LC. As shown in Figure 1b, LC could increase the cell viability of SH-SY5Y cells. In addition, the higher the concentration, the better effect LC will have. The IC50 of Loureirin C on SH-SY5Y cells was 4.942 μM.

#### 2.1.2. In Vitro Antioxidant Activities of LC

**Figure 2:**
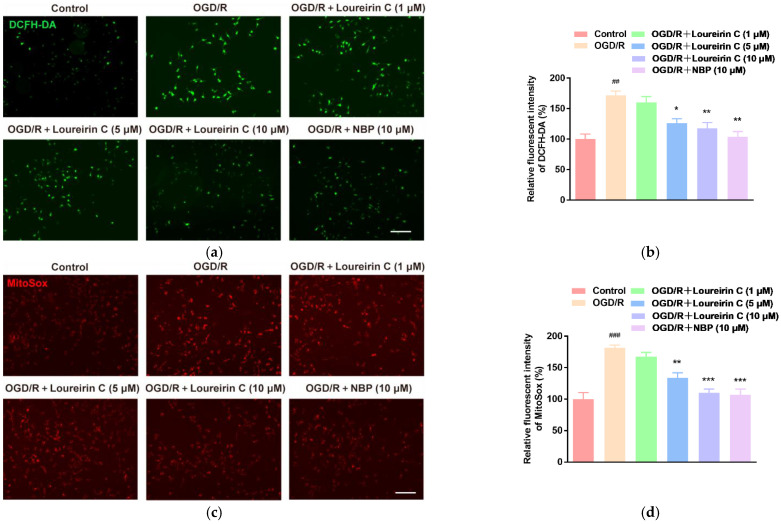
LC inhibits the release of ROS. (**a**) The SH-SY5Y cells with different treatments were stained with DCFH-DA (green fluorescence) and imaged by a laser scanning confocal microscopy. (**b**) Bar graph is the fluorescence intensity analyses of (**a**); (**c**) the SH-SY5Y cells with different treatments were stained with MitoSOX™ Red and imaged by a laser scanning confocal microscopy. (**d**) Bar graph shows the fluorescence intensity analyses of (**c**)**.** (Data are expressed as mean ± SD (*n* = 3). ### *p* < 0.001, ## *p* < 0.01 vs. Ctrl; *** *p* < 0.001, ** *p* < 0.01, * *p* < 0.1 vs. OGD/R group. Scale bar = 100 μm).

DCFH-DA can be used to label the intracellular ROS. As shown in Figure 2a, only negligible green fluorescence was observed in control group, which indicates less intracellular ROS existed. However, the green fluorescence enhanced in OGD/R group, which may be attribute to the generation of ROS. Moreover, compared with OGD/R group, the OGD/R+LC groups exhibited an obvious decreasing fluorescence, indicating that LC inhibited the generation of ROS or removed the generated ROS. The fluorescence intensity of DCFH-DA (Figure 2b) for OGD/R and OGD/R+LC (5, 10 μM) groups quantitatively verifies the results of confocal laser scanning microscopy. From Figure 2d, it can be found that the LC shows less intracellular ROS scavenging activity than the positive antioxidant drug butylphthalide (NBP). ROS can also be generated from the mitochondria. MitoSox^TM^ Red can be used to measure the ROS released from the mitochondria. To explore the effect of LC on the production of mitochondrial ROS, the SH-SY5Y cells after OGD/R treatment were exposed with 1, 5, or 10 μM LC. As shown in Figure 2c,d, OGD/R treatment induced high mitochondrial ROS production. LC (5, 10 μM) obviously inhibited or removed the mitochondrial ROS in SH-SY5Y cells after OGD/R treatment. From Figure 2d, it can be found that the mitochondrial ROS scavenging activity of 10 μM LC is comparable to the reference standards (NBP).

### 2.2. The Theoretical Studies of Free Radical Scavenging Mechanisms of LC

#### 2.2.1. The Determination of the Preferred Conformation of LC

**Figure 3:**
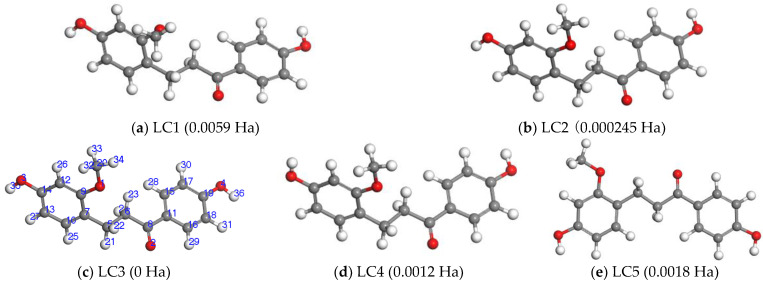
The optimized structures of LC in gas phase. (The numbering scheme for LC are shown in Figure 3c).

The initial structure of LC (LC1 in Figure 3a) was obtained from the NCBI Pubchem Compound database (http://www.ncbi.nlm.nih.gov/pccompound, CID: 14157896, accessed on 1 January 2021). LC is one of the main dihydrochalcones in Chinese dragon’s blood possessing two benzene rings joined by a linear three carbon chain. There is a methoxy group and a hydroxyl group conjugated with A ring, and there is a hydroxyl group conjugated with B ring. The three carbon chain between two aromatic rings contains an ethylene group and a carboxyl group. From Figure 3a, it can be found that two benzene rings of LC molecule are non-planar, the methoxy group is not in the plane of the aromatic ring A. In order to obtain the most stable structure of LC, five conformers of LC were considered. For the four conformers of LC1-LC4 in Figure 3, there are roughly the same dihedral angles (∠(C7-C9-C11-C15), −53.88° of LC3) between two benzene rings. The methoxy group of LC1 stretches forward out of the plane of the aromatic ring A, but those of LC2-LC5 are in the plane of the aromatic ring A. It can be seen from Figure 3 that the spatial orientations of the 2-OH group of LC2, LC3, LC4 are different. In order to find other possible stable conformers, LC5 conformer designed by a larger internal rotations between two aromatic rings, two benzene rings of LC5 conformer are almost in the same plane (dihedral angle ∠(C7-C9-C11-C15) is almost equal to zero). At the GGA-PBE/NDP4.4 level, the optimized structures of LC in gas phase together with their energies (Δ*E*_0_/Ha, 1 Ha = 27.212 eV, Δ*E*_0_ is the relative energy of each conformer with respect to the most stable LC3 conformer) are shown in Figure 3 and the structure parameters of LC3 are listed in Table 1. The results (see Table 1, the structure parameters of LC3 in water are shown in parentheses) show that LC3 (Figure 3c) has lowest energy. From this, we can come to the conclusion that LC has a twisted molecular structure with a dihedral angle ∠(C7-C9-C11-C15) of −53.88° (LC3 in Figure 3c), and the methoxy group is in the plane of the aromatic ring A. Therefore, in this work, LC3 was chosen out from their conformers for subsequent calculations.

#### 2.2.2. Fukui Function Analyses 

**Figure 4:**
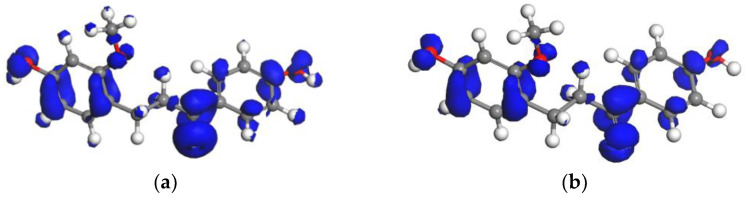
The Fukui function $f^{0}\left(r\right)$ of LC in gas phase (**a**) and in water (**b**) (mapped onto the isosurface of the total electron density, isovalue = 0.012507 a.u.).

For clarity, the Fukui function for radical attack $f^{0}\left(r\right)$ of LC molecule in gas phase and water was mapped onto the total electron density (Figure 4, isovalue = 0.012507 a.u.). From Figure 2, it can be found that the O atoms of the hydroxyl groups (O3, O4), the carbon atoms in two benzene groups (C7, C9, C10, C12, C13, C14 of A ring and C11, C15, C16, C17, C18, C19 of B ring) and the carbon and oxygen atoms in carbonyl groups (O2, C8) are all the reactive sites.

The condensed Fukui indices for free radical attack *f_k_*^0^(r) (see Table 2) for C, O atom in LC molecule were calculated based on the Hirshfeld charges, quantitatively verified the isosurface analysis results. These results revealed that hydroxyl, two benzene and carbonyl groups are the potential sites for the free radical attack. The carbonyl group (O2, C8) has the highest Fukui indices (see Table 2), implying that the carbonyl group (C=O group) is the more favorable site to be attacked by OH^•^ free radical. The results obtained in this study are supported by the previously published studies of flavonoid compounds and phenolic resin [26,27]. However, Cao et al. [28] found that Fukui function (FF) could not correctly predict the nucleophilic reaction sites for all aromatic molecules, which indicates that it is not completely reliable to predict the active place for radical scavenging by Fukui indices. Therefore, in this study, Fukui functions were only used as assistance for making predictions or explanations for the site reactivity and selectivity of loureirin C. The most active group of LC reactions with free radical will be determined by reaction enthalpy criteria (the reaction enthalpy studies will be given below).

In addition, from Figure 4 and Table 2, it can be observed that the chemical reactivity of LC molecule in water has the same changing rule as in gas phase. However, according to the Fukui indices, it is difficult to determine by what mechanisms the free radicals attack the LC molecule: by hydrogen abstraction, by radical adduct, or by some other mechanism.

#### 2.2.3. The Reactivity Analyses 

The scavenging of free radicals seems to play a notable part in the antioxidant activity of phenolic compounds. Five reaction mechanisms between phenolic compounds and reactive oxygen species (ROS, such as hydroxyl radical OH^•^) were summarized [29]: (1) direct hydrogen atom transfer (HAT); (2) sequential proton loss electron transfer (SPLET); (3) sequential electron-proton transfer or SEPT; (4) radical adduct formation; and (5) single-electron transfer (SET), where, (1), (2), and (3) all belong to hydrogen atom extraction mechanism. Thus, the scavenging radicals mechanisms are classified into three kinds, hydrogen atom extraction, radical adduct formation, and electron transfer. Rossi et al. [30] studied the hydroxyl radical scavenging mechanism of phenolic compounds, resveratrol, pterostilbene, and trimethoxystilbene. They found that the hydroxyl radical scavenging mechanism by e-transfer played a more important role than the H transfer mechanism for pterostilbene and trimethoxystilbene.

**Figure 5:**
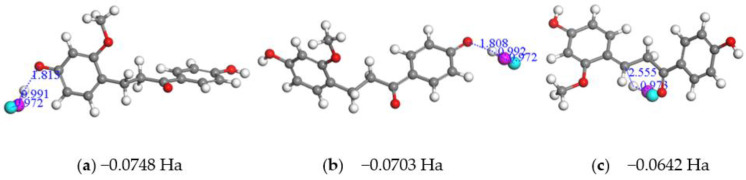

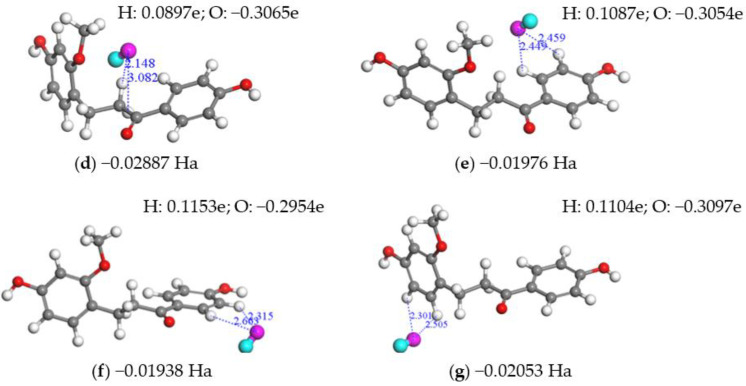
Optimized structures of LC+OH^•^ radical in gas phase; (**a**) near to the OH of A ring (left) of LC; (**b**) near to the OH of B ring(light) of LC; (**c**) near to H21; (**d**) near to H23 and H24; (**e**) near to H28 and H30; (**f**) near to H29 and H31; and (**g**) near to H25 and H27. The energy difference between the LC+OH^•^ complex and two reactants and the distances (Å) between the oxygen atom of OH^•^ and the hydrogen atom of LC, between the hydrogen atom of LC and its neighbor C, and between the two atoms of OH^•^-radical are shown. The Hirshfeld charges of O and H atoms of OH^•^ are shown at the top of Figure 5 (**d**–**g**).

In order to determine the attack mechanism of free radicals to LC, we added an OH^•^ radical near to the hydrogen atoms of the LC, and constructed 7 initial structures for the LC+OH^•^ radical complexes. Figure 5 shows the structures optimized in vacuum, and those in water are shown in Figure S1 (Supplementary Materials). From Figure 5, it can be found that when the OH^•^ radical was added near to H atoms (H35, H36) of the two hydroxyl groups and to H21 atom of three carbon chain (C5-C6-C8, which joins two benzene rings of LC, see Figure 5a–c), the OH^•^ radical can abstract a hydrogen atom from LC and changes into a H_2_O molecule.

In contrast, the other hydrogen atoms of LC cannot be abstracted by the OH^•^ radical, as indicated in Figure 5d–g. The distances (Å) between the oxygen atom of OH^•^ and the hydrogen atom of LC in Figure 5d–g are longer than those in Figure 5a–c, which suggests that these interactions between OH^•^ radical and LC molecule (Figure 5d–g) are weaker. In addition, the Hirshfeld charges of O and H atoms of OH^•^ are shown in Figure 5d (such as H: 0.0897e; O: −0.3065e), (e), (f), (g); from these values, it can be found that the OH^•^ free radical gains electrons (Figure 5d, 0.2168e), which suggests the transfer of electrons from the LC towards the OH^•^ radical. Accordingly, the electrostatic attraction generated by e-transfer binds free radicals to LC molecules and this may be a mechanism of LC molecules scavenging free radicals. The three structures (Figure 5a–c) are more stable than the other four optimized structures from an energy point of view (see the energy difference between the LC+OH^•^ complex and two reactants). Therefore, we can conclude that the OH* radical prefers to attack to the hydroxyl groups and H21 of LC by hydrogen abstraction mechanism. From Figure S1, it can be found that water does not change the sites most likely to be attacked by free radicals. Comparing Figure 5 with Figure S1, all structures in water are more stable than those in gas phase, indicating that the dehydrogenation is largely preferable in water.

**Figure 6:**
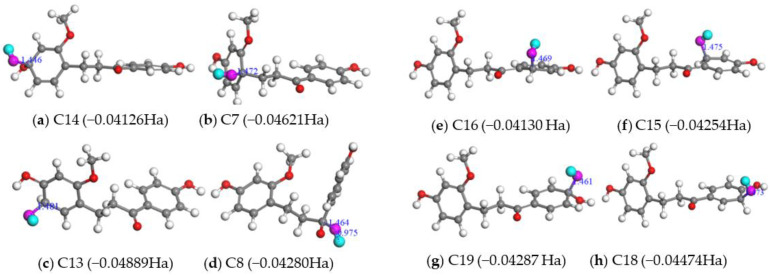
Optimized structures of the radical adducts of LC+OH^•^ in gas phase. OH^•^ was added on the C14 (**a**), C7 (**b**), C13 (**c**) sites of LC (A ring, left); on the site of H8 of the C=O double bond (**d**); on the C16 (**e**), C15 (**f**), C19 (**g**), C18 (**h**) sites of LC (B ring, right). The energy difference between the LC+OH^•^ adduct and two reactants and the distances (Å) between the oxygen atom of OH^•^ and the C atom of LC are shown.

As far as RAF mechanism, we considered two kinds of RAF channels: one is that OH^•^ radical was added to the C atom on site C8 of the carbonyl groups (C=O group), the other is that OH^•^ radical was added to the C atoms of two benzene rings of LC. The optimized structures reflecting RAF mechanism in gas phase and in water are depicted in Figure 6 and Figure S2 (Supplementary Materials). From Figure 6 and Figure S2, it can be found that when the OH^•^ radical was added to the different sites of LC molecule, all radical adducts formed by Equation (3) were with lower energies relative to the total energy of the reactants Equation (3), which indicated that RAF mechanism is the one mechanism of LC molecule to remove OH^•^ radicals either in gas phase or in water from an energy point of view.

### 2.3. The Free Radical Scavenging Mechanisms of LC via Hydrogen Atom Abstraction Mechanism

#### 2.3.1. Free Radical Scavenging Capacity via HAT

Reaction enthalpies BDEs in the gas phase involved in HAT radical scavenging mechanisms of LC are presented in Table 3. By comparing the reaction enthalpy, the thermodynamic preferred reaction pathway can be determined. From Table 3, it can be seen that the BDEs (C-H, O-H) of LC vary from 77.3364 to 111.1434 kcal/mol in gas phase. The phenolic OH group O3-H35 has the lowest BDE value of 77.3364 kcal/mol, followed by the O4-H36 (79.8971 kcal/mol), which indicates that phenolic OH groups have the greatest capacity to donate H. In addition, the -CH2-CH2-moiety between two aromatic rings of LC possess lower BDE values (lower than 90 kcal /mol, being 84.1793, and 85.4831 kcal/mol, respectively), which suggests C-H bonds of -CH2-CH2-moiety in LC have certain capacity to donate H atoms. From Table 3, it can be found that the H atoms of A and B aromatic rings are difficult to dissociate in the gas phase. The BDEs of LC in water are calculated and listed in Table 4. As can be seen from it, the solvent effect does not lead remarkable change to all BDE values. For example, the BDEs of O3-H35 calculated in the gas phase and in water are 77.3364 and 74.5010 kcal/mol, respectively. This means that the polar environment does not have much effect on direct hydrogen atom transfer (HAT). In the calculation formula (5) of BDE, H(H^•^) is the enthalpy of H^•^. From Table 5, it is found that the solvation enthalpy of H^•^ in water is −4 kJ/mol, which indicates the difference of the enthalpy of H^•^ is very small between in gas and in water. This is why the BDE values have little difference in gas and in water.

#### 2.3.2. Free Radical Scavenging Capacity via SEPT

The IP and PDE values reflecting SEPT mechanism for O-H and C-H bonds of LC were also calculated in both the gas-phase and water (data in Table 3 or Table 4). It can be seen from Table 3 that the changing rule of PDE in gas-phase is the same as that of BDE, that is, phenolic OH group O3-H35 has the lowest PDE value, which indicates that the O3-H35 phenolic OH group is the easiest deprotonation position. The IP value (95.9667 kcal mol^−1^) of LC in gas-phase is higher than the BDE values of phenolic OH groups and C-H groups in -CH2-CH2-moiety; and the PDE values for all studied bonds of LC in gas-phase are all much larger than the BDE values of the corresponding bonds of LC. These results suggest that SEPT mechanism (compared to the HAT mechanism) is not the preferred one. From Table 4, it is found that the solvent (water) has great influence on IP and PDE values. In the calculation Formula (9) of PDE, H(H^+^) is the enthalpy of proton. From Table 5, it is found that the solvation enthalpy of proton in water is −1090 kJ/mol, which indicates there is a large difference of the enthalpy of proton between in gas and in water. This is why the PDE values have great difference in gas and water. IP and PDE values in water are all lower than that BDE values, which indicates that in comparison to the HAT mechanism, the SEPT mechanism is the preferred one in water.

#### 2.3.3. Free radical Scavenging Capacity via SPLET

The calculated PA values of all present OH, C-H groups are all above 336 kcal/mol (as seen in Table 3), indicating proton transfer (the first step of SPLET) in gas-phase is more difficult comparing to HAT (the maximum of BDE is 111.1434 kcal/mol). Therefore, although all ETE values (the second step SPLET, electron transfer) are less than the minimum BDE value (<58 kcal/mol), the radical scavenging of LC via SPLET does not easily take place due to the high PA values. In addition, PA values of O-H, C-H groups are all higher than the corresponding PDE values, which means that the SPLET process more easily takes place than the SEPT process. From Table 3, it can be found that PA values in the gas phase are all higher than IPs (the first step of SEPT), indicating the radical scavenging of LC via SPLET more difficultly takes place compared with the SEPT. It can be observed that the ETEs in the gas phase (displayed in Table 3) are lower than IPs (data in Table 3). For example, the ETE of O3-H35 in the gas phase is 49.1445 kcal/mol, while the IP is 95.9667 kcal/mol. This means that the electron transfer from anionic form is more favorable than that from neutral one. In the meantime, when calculating in water, a dramatic decrease in PA values by comparison with the values calculated in the gas phase can be observed. For example, the PA value of O3-H35 of LC in the gas phase is 342.5314 kcal/mol, while its value in water is −17.8594 kcal/mol (see Table 4). The reason for such a dramatic decrease in PA values is that the solvation enthalpy of proton in water is very low compared to that in the gas phase (Δ_solv_*H* = −1090 kJ/mol, see Table 5). From Table 4, it can be observed that the PA values are all lower than the BDE, IP, and PDE values in water. Compared with HAT and SEPT mechanism, proton transfer (the first step of SPLET) is most likely to occur. Thus we propose that the hydrogen abstraction process depends on the ETE values (the second step SPLET, electron transfer). From Table 4, it can be seen that the ETE values of phenolic H groups are higher than the corresponding BDE values, while other ETE values, especially, the one of C5-H21, are smaller than corresponding BDE values; this indicates that in water SPLET mechanism is a preferred one in water in comparison with the HAT mechanism. Comparing the ETE values with the corresponding PDE values and IP value, it can be seen that the minimum value of ETE (55.0132 kcal/mol) is higher than the minimum value of PDE (40.2800 kcal/mol) and IP value (32.6024 kcal/mol), which suggests that in water SEPT mechanism is the favorite.

#### 2.3.4. The Antioxidant Mechanism for LC

Loureirin C is a dihydrochalcone (and a flavonoid) which is rich in dragon’s blood. Amic et al. [31] found that flavonoids are capable of eliminating ROS by scavenging free radicals, chelating metal ions, inhibiting prooxidant enzymes, activating antioxidants, and detoxifying enzymes. Burda et al. [32] found that the scavenging of free radicals seems to play a notable part in the antioxidant activity of flavonoid compounds. In this work, we found that LC increased the viability of SH-SY5Y cells treated with OGD/R (the model of ischemic stroke). We proposed the mechanism of action of LC involves its antioxidant properties. By (DCFH-DA) and MitoSox Red experiments, we demonstrated that the antioxidant action of LC is correlated to the eliminating of the intracellular/mitochondrial ROS. Our DFT studies supported the biological results. The mechanism of antioxidant of LC can be elucidated from Fukui function and reactivity analyses as well as the relative BDE, IP, PDE, PA, and ETE values. It is clear that H-abstraction reaction is the favorite antioxidant mechanism, and the O-H35 group is the most feasible site to be attacked by OH^•^. The HAT mechanism is dominant in gas-phase and in water, but the SEPT mechanism is the favorite hydrogen abstraction mechanism, and the O-H35 hydroxyl group has the greatest ability to donate H-atom.

## 3. Methods of Experiment and Calculation

### 3.1. LC Preparation 

The preparation method of LC is as follows. Briefly, the red resin (1.0 kg) of *Dracaena cochinchinensis* was grounded and extracted with ethyl acetate (EtOAc). The EtOAc extract (300 g) was passed through a silica gel column and eluted with petroleum/acetone to give six fractions as Fr.1-6. Subfractions of Fr.3, Fr.3-1, Fr.3-2, Fr.3-3, and Fr.3-4 were separated by repeated silica gel column chromatography (CH_2_Cl_2_/MeOH (methanol). LC was isolated from fraction Fr.3-1 by high performance liquid chromatography (HPLC) [33]. The chemical structure of LC is shown in Figure 3c. The IC50 and chromatograms of LC were shown in Figures S3 and S4 (Supplementary Materials).

### 3.2. Cell Culture

SH-SY5Y human neuroblastoma cells were purchased from American Type Culture Collection (ATCC, Product No. CRL-2266). SH-SY5Y cells were cultured in Dulbecco’s Modifed Eagle Medium (DMEM) supplemented with 10% fetal bovine serum, 2 mM d-glutamine, 100 U/mL penicillin, and 100 μg/mL streptomycin at 37 °C in a humidifed incubator containing 5% CO_2_ [34]. For OGD/R treatment, SH-SY5Y cells were first incubated in glucose-free DMEM, and were subsequently transferred into a hypoxia incubator (Heal Force, HF100) with 1% O_2_, 94% N_2_, and 5% CO_2_ for 6 h at 37 °C. After OGD treatment, SH-SY5Y cells were incubated in the DMEM with or without LC for another 24 h in the incubator with 95% air and 5% CO_2_.

### 3.3. Cell Viability Assay

Cell viability was evaluated by a MTT (Sigma, St. Louis, MO, USA) assay. Briefly, the SH-SY5Y cells were seeded in 100 μL DMEM at a density of 6000 cells/well in 96-well plates. After OGD/R treatment, the cells were incubated with MTT solution (0.5 mg/mL) for 4 h at 37 °C. Then, we discarded the MTT solution and added 150 μL dimethyl sulfoxide (DMSO) in each well. The optical density (OD) of the absorbance of the wells was measured at a wavelength of 490 nm.

### 3.4. DCFH-DA Staining

The SH-SY5Y cells were seeded in 24-well plates with 500 μL DMEM at a density of 2 × 10^4^ cells per group. After OGD/R treatment, PBS was used to wash the cells for 5 min. Then, the cells were fixed with PFA for 30 min and washed with phosphate buffered saline (PBS) once again. The cells were incubated with 10 µM DCFH-DA (Beyotime, Shanghai, China), a fluorescent probe for ROS, and dyed for 30 min. Finally, the cells were washed with PBS three times. Fluorescence microscopy was employed to acquire the images of SH-SY5Y cells, and the fluorescence pictures were analyzed by Image-Pro Plus 5.0 software (Media Cybernetics, Inc., Rockville, MD, USA).

### 3.5. MitoSox Staining

MitoSOX Red is a mitochondrial superoxide probe. We used MitoSOX Red (Invitrogen, Carlsbad, CA, USA) staining to analyze the mitochondrial production of reactive oxygen species (ROS). The SH-SY5Y cells were seeded in 24-well plates with 500 μL DMEM at a density of 2 × 10^4^ per group. After OGD/R, 10 µM MitoSOX Red was added to each well and the cells were incubated for 30 min at 37 °C in the dark. After incubation, the cells were washed with PBS twice, then the samples were analyzed by fluorescence microscopy to identify SH-SY5Y-positive cells (red staining).

### 3.6. Computational Methods

All the calculations are performed using the Dmol3 module of MS6.0 commercial software [35]. The generalized gradient approximation (GGA) with the Perdew–Burke–Ernzerhof (PBE) functional was employed to describe the exchange-correlation potential [36]. To obtain a better description of the interaction of LC and OH^•^ radical, the newly implemented Grimme corrections (DFT-D) were applied with a s6 factor of 0.75 and a damping factor d of 20.0. We adopted the double numerical polarized (DNP) basis set as the basis set, because the DNP basis set (version 4.4) [37] is comparable with the Gaussian 6-31G (d, p) basis set and exhibits a better accuracy. In order to achieve better energy convergence, an orbital cutoff of 0.37 nm, a Fermi smearing of 0.008 Ha to the orbital occupation, and the density mixing fraction of 0.2 with direct inversion in the iterative subspace (DIIS size 6) were used. Spin unrestricted approach and the asymmetry were used. In these calculations, for the neutral LC molecule, the spin multiplicity is set to 1. For LC radical anion (LC^•−1^) and LC cation, the spin multiplicity is set to 1 and 2, respectively. LC radicals and LC-hydroxyl radical complexes were considered to have zero charge and multiplicity 2. The convergence criteria is 1.0 × 10^−5^ Ha for energy, 0.004 Ha/Å for force, and maximum displacement is 0.005 Å for displacement in the geometry optimization. It is well known that drugs work in the cellular environment, so we should take into account the influence of solvents when we investigate the antioxidant mechanism of LC in cellular environments by scavenging the free radicals. In this work, solvent effects are introduced using the continuum solvation model (COSMO) [38]. In the cell, water is the major component, although the dielectric constant of water is somewhat larger than that of cellular environments. In this work, water was used as solvent to simulate the internal environments of body. The local minima nature was confirmed by frequency calculations. The stable species show positive real values for all of the vibrational frequencies. Total reaction enthalpies of the studied species, *H*, were calculated at 298.15 K using the following formula [39]:

*H* = *E*_0_ + *ZPE* + *H*_trans_ + *H*_rot_ + *H*_vib_ + RT (1)

where *E*_0_ is the total electronic energy at 0K, *ZPE* is the zero-point vibrational energy. *H*_trans_, *H*_rot_, and *H*_vib_ are contributions from translational, rotational, and vibrational degrees of freedom to the enthalpy. RT represents PV-work term contribution to enthalpy.

It is generally assumed that a phenolic compound follows two basic types of mechanisms for the radical scavenging: (1) Hydrogen atom abstraction; (2) radical adduct formation. LC is a phenolic compound, so the radical scavenging mechanisms for LC can be described by:

Hydrogen atom abstraction:

LC + OH^•^→ (LC–H)^•^ + H_2_O (2)

Radical adduct formation:

LC + OH^•^→ LC(OH)^•^ (RAF) (3)

It is well known that hydrogen atom abstraction involves complex processes. At present, there are three different mechanisms that are commonly accepted: (1) direct hydrogen atom transfer (HAT); (2) sequential electron-proton transfer (SEPT); (3) sequential proton loss electron transfer (SPLET). 

In HAT mechanism, a hydrogen atom (H^•^) of LC is transferred to OH^•^ radical:

LC → (LC–H)^•^ + H^•^ (4)

This reaction process is controlled by the bond dissociation enthalpy (BDE) of OH or CH bond of LC, the BDE is calculated according to:

BDE = *H*((LC–H)^•^) + *H*(H^•^) − *H*(LC) (5)

In the SEPT mechanism, an electron from LC is transferred to OH^•^ radical firstly, and then LC radical cation (LC^+•^) releases a proton. The reaction processes are as follows:

LC → LC^+•^ + e^−^ (6)

LC^+•^ → (LC–H)^•^ + H^+^ (7)

The reaction mechanism of Equation (6) is governed by the ionization potential (IP), while that for Equation (7) can be characterized by the proton dissociation enthalpy (PDE). IP and PDE are calculated as follows:

IP = *H*(LC^+•^) + *H* (e^−^) − *H*(LC) (8)

PDE = *H*(LC–H)^•^) + *H*(H^+^) − *H*(LC*^+^*^•^) (9)

In the SPLET mechanism, the first step is the deprotonation of LC molecule, and then, LC-H (deprotonation) anion gives an electron to OH^•^:

LC → (LC–H)^−•^+ H^+^ (10)

(LC–H)^−•^ →(LC–H)^•^ + e^−^ (11)

The related proton affinity (PA) and electron transfer enthalpy (ETE) in the above steps can be calculated as follows:

PA = *H*((LC–H)^−^) + *H*(H^+^) − *H*(LC) (12)

ETE = *H*(LC–H)^•^ + *H*(e^−^) − *H*((LC–H)^−•^) (13)

In the equations above, *H*(H^•^), the enthalpy of hydrogen atom in gas-phase, is −0.4979 Ha [40,41,42]; *H*(H^+^), the gas phase enthalpy of proton, is 6.197 kJ /mol, and *H*(e^−^) is enthalpy of electron(e^−^) in gas-phase with 3.145 kJ/mol [41]. Solvent contribution to the enthalpies of *H*(H^•^), *H*(H^+^) and *H*(e^−^) in water are collected in Table 5.

Fukui functions were calculated at GGA-PED/NDP (4.4 Version) levels, because it is preferable for the reactivity analysis to be regional [35]. To calculate $f^{0}\left(r\right)$ (Fukui function for free radical attack) [43], the wave functions of these model molecules with neutral (Q = 0), cationic (Q + 1) and anionic (Q − 1) charges were also obtained.

(14) $$f^{0}\left(r\right)=\left[\rho_{N+1}\left(r\right)-\rho_{N+1}\left(r\right)\right]/2\;$$

(15) $$f_{k}^{0}=\left[q_{k}\left(N+1\right)-q_{k}\left(N-1\right)\right]/2$$

where $f^{0}\left(r\right)$ denotes the Fukui function for free radical attack of a molecule with N electrons, *f*_k_^0^ denotes the Fukui index for free radical attack of atom k; *ρ*_N+1_(r) and *ρ*_N−1_(r) denote electron density of a molecule with N+1 and N−1 electrons, respectively; *q*_k_(N+1) and *q*_k_(N−1) denote atomic charge of atom k in molecule with N+1 and N−1 electrons, respectively.

## 4. Conclusions

In this work, we have investigated the cytotoxicity and the protective antioxidant activity of the LC. The results show that LC has almost no effect on cell viability under 15 μM, and it increases the viability of SH-SY5Y cells after OGD/R treatment. The results of DCFH-DA and MitoSox Red experiments indicate LC is very efficient in inhibiting the generation of the intracellular/mitochondrial ROS or removing these two kinds of ROS generated. Fukui function analyses suggest three radical scavenging mechanisms of LC: hydrogen abstraction mechanism, the complex formation by e-transfer, and RAF mechanism. The smallest energy difference between the product and two reactants of the attack of OH^•^ to LC in H-abstraction reactions (−0.0748 Ha, OH^•^ near to H35) is lower than that in the complex formation by e-transfer (−0.02887 Ha, OH^•^ near to H23 and H24) and RAF mechanism (−0.04889 Ha, OH^•^ adds to C13), which demonstrates that H-abstraction reaction is the most favorite mechanism, and the O-H35 group is the most feasible site to be attacked by OH^•^. For hydrogen abstraction mechanism, HAT, SPLET, and SEPT mechanisms were studied in gas phase and in water by the bond dissociation enthalpies (BDE), proton affinities (PA), ionization potential (IP), proton dissociation enthalpy (PDE), and electron transfer enthalpy (ETE). In water, IP (32.6024 kcal/mol) and the lowest PDE (40.2800 kcal/mol of O3-H35) value are lower than the lowest BDE (74.5010 kcal/mol of O3-H35) value, indicating that the SEPT mechanism is a preferred one in water in comparation with the HAT mechanism. The PA value of O3-H35 of LC in water is −17.8594 kcal/mol, thus the first step of SPLET would occur spontaneously. The minimum value of ETE (55.0132 kcal/mol) is higher than the minimum value of PDE (40.2800 kcal/mol of O3-H35) and IP value (32.6024 kcal/mol), which suggests that SEPT mechanism is a preferred one in water in comparison with the SPLET mechanism. Therefore, we can draw a conclusion that the SEPT mechanism is the most favored hydrogen abstraction mechanism in water, and the O-H35 hydroxyl group has the greatest ability to donate H-atoms.

## Figures and Tables

**Table 1:** Structure parameters of different LC conformation (bond length/Å, bond angle, and dihedral angle/°).

Parameter	LC1	LC2	LC3	LC4	LC5
*O3-H35*	0.973	0.973	0.973 (0.976)	0.973	0.973
*O3-C14*	1.379	1.381	1.380 (1.380)	1.381	1.381
*O4-H36*	0.974	0.974	0.974 (0.977)	0.974	0.974
*O4-C19*	1.373	1.373	1.372 (1.367)	1.373	1.372
*∠C14-O3-H35*	107.898	107.662	107.696 (108.149)	108.504	107.586
*∠C19-O4-H36*	108.248	108.233	108.16 (108.838)	108.297	108.194
*∠C9-C7-C11-C15*	−58.971	−54.396	−53.880 (−61.099)	−59.786	178.021
*ΔE_0_/(Ha)*	0.006	0.001	−919.487 (−0.026)	0.006	0.002

**Table 2:** The Fukui indices $f_{k}^{0}$ of atoms of LC in gas phase and in water (atomic labels in LC, see Figure 3c).

Atom	O1	O2	O3	O4	C5	C6	C7	C8	C9	C10
$f_{k}^{0}$ in gas	0.021	0.102	0.052	0.045	0.008	0.016	0.035	0.068	0.021	0.023
$f_{k}^{0}$ in water	0.034	0.094	0.049	0.032	0.011	0.018	0.050	0.076	0.030	0.029
Atom	C11	C12	C13	C14	C15	C16	C17	C18	C19	C20
$f_{k}^{0}$ in gas	0.021	0.021	0.048	0.037	0.031	0.035	0.031	0.031	0.047	0.011
$f_{k}^{0}$ in water	0.028	0.023	0.057	0.040	0.035	0.038	0.026	0.025	0.041	0.011

**Table 3:** The calculated parameters of free radical scavenging activity for LC (in kcal/mol) in gas-phase.

	HAT	SEPT		SPLET	
	BDE	IP	PDE	PA	ETE
		95.9667			
O3-H35	77.3364		295.7091	342.5314	49.1445
O4-H36	79.8917		298.2644	336.9088	57.3223
C16-H29	110.5625		328.9352	397.7635	27.1384
C18-H31	107.6919		326.0646	384.6286	37.4027
C17-H30	110.2604		328.6331	391.9849	32.6149
C15-H28	106.7397		325.1124	392.4227	28.6564
C6-H23	85.4831		303.8558	362.1344	37.6881
C5-H21	84.1793		302.5520	374.1161	24.4026
C10-H25	108.5907		326.9634	391.8397	31.0904
C13-H27	110.1209		328.4936	385.6423	38.8180
C12-H26	111.1434		329.5161	384.1790	41.3038

**Table 4:** The calculated parameters of free radical scavenging activity for LC (in kcal/mol) in water.

	HAT	SEPT		SPLET	
	BDE	IP	PDE	PA	ETE
		32.6024			
O3-H35	74.5010		40.2800	−17.8594	90.7418
O4-H36	75.3943		41.17328	−19.1905	92.9662
C16-H29	106.8953		72.6743	26.0949	79.1818
C18-H31	110.8465		76.6255	27.7661	81.4618
C17-H30	110.3096		76.0886	27.6415	81.0495
C15-H28	107.9299		73.7089	30.0925	76.2188
C6-H23	82.5412		48.3202	−0.6891	81.6117
C5-H21	79.3396		45.1186	22.7078	55.0132
C10-H25	108.4034		74.1824	34.6772	72.1076
C13-H27	110.4871		76.2661	32.4688	76.3997
C12-H26	110.7761		76.5551	27.6081	81.5494

**Table 5:** Solvation enthalpies in water of H^•^, H^+^, and e^−^ in kJ/mol.

	H^•^	H^+^	e^−^
Δ_solv_*H* [31]	−4	−1090	−236

## Data Availability

Data are available from the corresponding authors upon request.

## Supplementary Materials

The following supporting information can be downloaded at: https://www.mdpi.com/article/10.3390/molecules28010380/s1, Figure S1: Optimized structures of LC + OH^•^ radical in water; (a) near to the OH of A ring (left) of LC; (b) near to the OH of B ring(light) of LC; (c) near to H21; (d) near to H23 and H24; (e) near to H28 and H30; (f) near to H29 and H31 and (g) near to H25 and H27. The energy difference between the LC+OH* adduct and two reactants and the distances (Å) between the oxygen atom of OH^•^ and the hydrogen atom of LC, between the hydrogen atom of LC and its neighbor, C, and between the two atoms of OH^•^ radical are shown. The Hirshfeld charges of O and H atoms of OH^•^ are showed at the top of Figure S1 (d), (e), (f), (g); Figure S2: Optimized structures of LC+OH^•^ in water. OH^•^ was added on C14 (a), C7 (b), C13 (c) sites of LC (A ring, left); on the site of C8 of the C=O double bond (d); on the C16 (e), C15 (f), C19 (g), C18 sites of LC (B ring, right) (h). The energy difference between the LC+OH* adduct and two reactants and the distances (Å) between the oxygen atom of OH^•^ and the C atom of LC are shown; Figure S3: IC50 of LC; Figure S4: Chromatograms of LC.
