# Supplementary material for: Theoretical and Experimental Investigation of the Antioxidation Mechanism of Loureirin C by Radical Scavenging for Treatment of Stroke
Source: Molecules. 2023 Jan 2;28(1):380. doi: 10.3390/molecules28010380 (PMC9822359; doi:10.3390/molecules28010380)

Supporting Materials

Theoretical and Experimental Investigation of the Antioxidation Mechanism of Loureirin C by Radical Scavenging for Treatment of Stroke

Ye-Shu Liu[1,2], Guo-Ying Zhang[3,*] and Yue Hou[1,2,*]

[1]College of Life and Health Sciences, National Frontiers Science Center for Industrial Intelligence and Systems Optimization, Northeastern University, Shenyang, 110819 China
[2] Key Laboratory of Data Analytics and Optimization for Smart Industry, Northeastern University, Ministry of Education, Shenyang, 110819 China
[3]College of Physics Science and Technology, Shenyang Normal University, Sheyang, 110034 China.
[*]Correspondence: gyzhang1965@sina.com or zhanggy@synu.edu.cn (G.-Y.Z.); houyue@mail.neu.edu.cn (Y.H.)

Figure S1. Optimized structures of LC+OH$^{•}$ Pradical in water; (a) near to the OH of A ring (left) of LC; (b) near to the OH of B ring(light) of LC; (c) near to H21; (d) near to H23 and H24; (e) near to H28 and H30; (f) near to H29 and H31 and (g) near to H25 and H27. The energy difference between the LC+OH$^{•}$ complex and two reactants and the distances (Å) between the oxygen atom of OH$^{•}$ and the hydrogen atom of LC, between the hydrogen atom of LC and its neighbor, C, and between the two atoms of OH$^{•}$ radical are shown. The Hirshfeld charges of O and H atoms of OH$^{•}$ were showed at the top of Fig. S1 (d), (e), (f), (g).

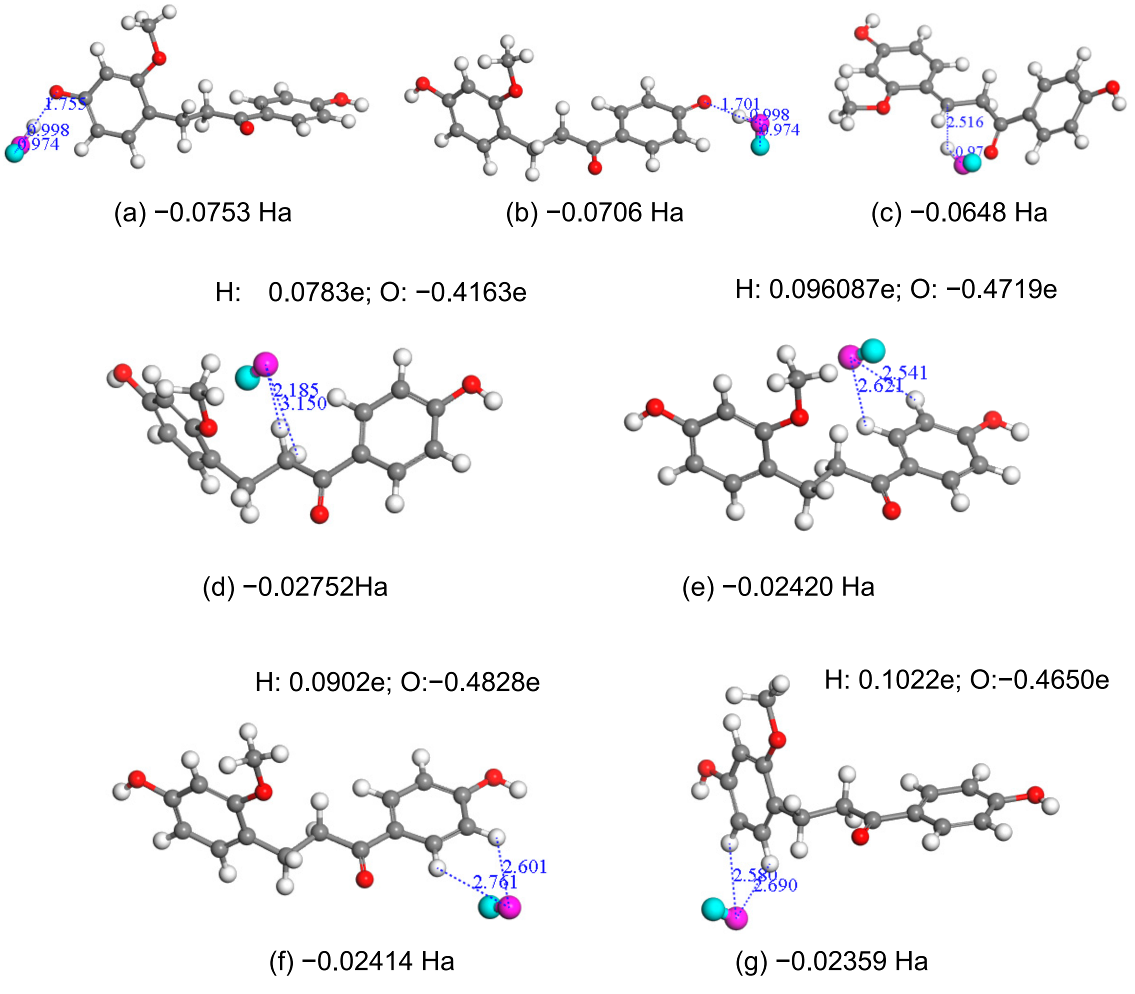

Figure S2: Optimized structures of LC+OH$^{\bullet}$ in water. OH$^{\bullet}$ was added on C14(a), C7(b), C13(c) sites of LC (A ring, left); on the site of C8 of the C=O double bond (d), on the C16(e), C15(f), C19 (g), C18(h) sites of LC (B ring, right). The energy difference between the LC+OH$^{\bullet}$ adduct and two reactants and the distances (Å) between the oxygen atom of OH$^{\bullet}$ and the C atom of LC are shown.

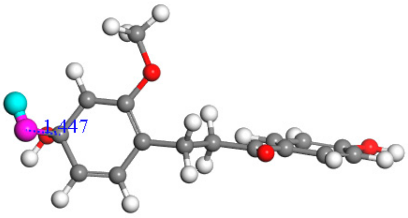

(a) C14+OH$^{\bullet}$(−0.0397 Ha)

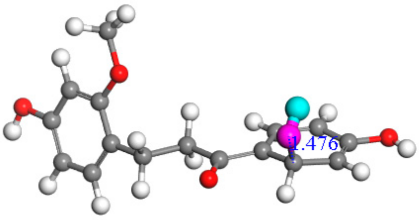

(b) C16+OH$^{\bullet}$(−0.0406 Ha)

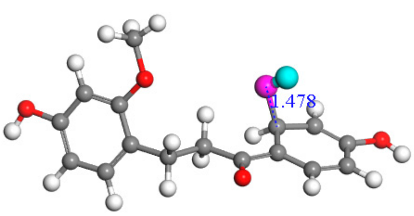

(c) C15+OH$^{\bullet}$(−0.0399 Ha)

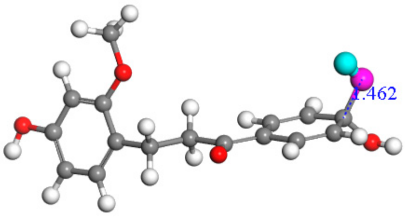

(d) C19+OH$^{\bullet}$(−0.03999 Ha)

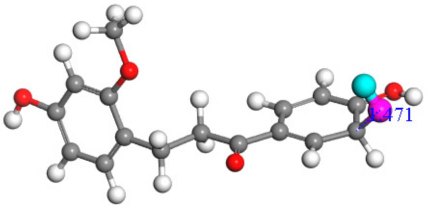

(e) C18+OH$^{\bullet}$(−0.03886 Ha)

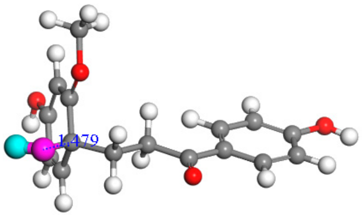

(f) C7+OH$^{\bullet}$(−0.04576 Ha)

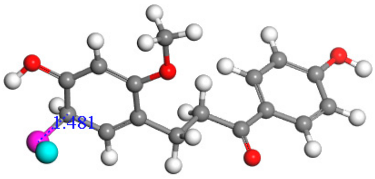

(g) C13+OH$^{\bullet}$(−0.04370 Ha)

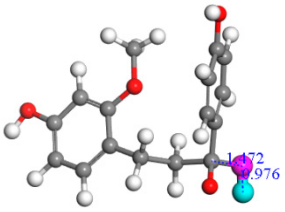

(h) C8+OH$^{\bullet}$(−0.04145 Ha)

Figure S3: IC50 of LC

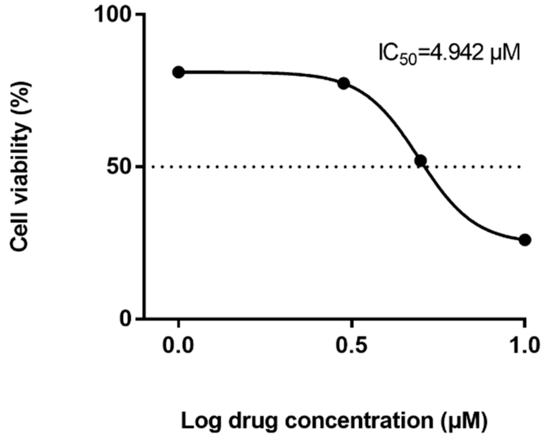

Figure S4：Chromatogram of LC

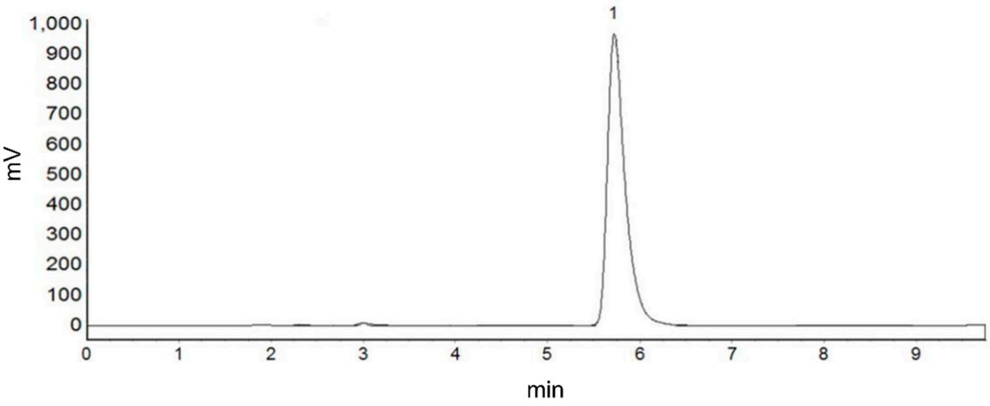

Supplement: Supplementary file 1 [file molecules-28-00380-s001.zip › molecules-1979638-supplementary.pdf]
